# Predictive breeding and marker-assisted selection for grain quality and freezing tolerance in durum wheat

**DOI:** 10.3389/fpls.2026.1739121

**Published:** 2026-03-10

**Authors:** Yawar Habib, Giuseppina Angione, Paolo Vitale, Hassan Baneh, Vincenzo Natoli, Concetta Lotti, Svetlana D. Dolaberidze, Liudmila A. Bespalova, Alexandra A. Mudrova, Aleksey S. Ianovskii, Salvatore Esposito, Pasquale De Vita

**Affiliations:** 1 Project Centre for Agro Technologies, Skolkovo Institute of Science and Technology, Moscow, Russia; 2 Research Centre for Cereal and Industrial Crops (CREA-CI) - Council for Agricultural Research and Economics, Foggia, Italy; 3 Department of Agriculture, Food, Natural Science, University of Foggia, Engineering, Foggia, Italy; 4 International Maize and Wheat Improvement Center (CIMMYT), Texcoco, Estado. de México, Mexico; 5 Animal Science Research Department, Kurdistan Agricultural and Natural Resources Research and Education Center, Agricultural Research, Education and Extension Organization (AREEO), Sanandaj, Iran; 6 Genetic Services SRL, Deliceto, Italy; 7 Agroliga Plant Selection Center Ltd, Moscow, Russia; 8 Federal State Budgetary Scientific Institution “NTsZ im. P.P. Lukyanenko”, Krasnodar, Russia; 9 Institute of Biosciences and Bioresources (CNR-IBBR), Research Division, Portici, Italy

**Keywords:** freezing tolerance, grain quality, GWAS, KASP, MTAs, parental selection

## Abstract

Durum wheat, a globally significant crop for high-quality pasta production, remains vulnerable to unseasonal freezing events, a risk that is intensified with climate variability. To address this challenge, we combined genome-wide association studies (GWAS), genomic prediction, and marker-assisted selection to improve both freezing tolerance and grain quality in durum wheat. A panel of 250 diverse accessions, comprising cold-adapted lines from Eastern Europe and high-quality genotypes from Southern Europe, was genotyped using a 25K SNP array. Clear genetic differentiation by geographical origin and growth habit highlighted contrasting allelic patterns for adaptation and quality traits. Phenotypic evaluations were carried out in experimental field trials over two consecutive growing seasons in Italy and Russia to assess the freezing tolerance and quality performance of the genetic materials. GWAS identified five significant marker-trait associations (MTAs) for freezing tolerance on chromosomes 2A, 2B, 3B, 4A, and 5A. Notably, a strong MTA on chromosome 5A (physical position 488.2 Mb) individually explained up to 27% of the phenotypic variance (PVE), co-localizing with the critical *Fr-A2* cold-stress regulatory locus. Significant associations for grain-quality traits were localized on a 1B chromosome hotspot (541–652 Mb). A multi-trait genomic selection model integrating freezing tolerance, grain weight, and gluten traits enabled the identification of optimal parental lines, resulting in measurable gains across simulated generations. From the top-ranked crosses, BC_2_F_2_ populations were developed and genotyped with KASP markers targeting validated MTAs. Lines carrying favorable alleles for both freezing tolerance and gluten strength were successfully selected, confirming the predictive accuracy of the model. The integration of GWAS, diversity-preserving genomic prediction, and functional marker validation offers a robust and scalable pipeline for breeding cold-resilient, high-quality durum wheat, providing tangible tools to adapt Mediterranean and similar wheat systems to increasing climate variability.

## 1 Introduction

Durum wheat (*T. turgidum* L. subsp*. durum* (Desf.) Husn.) is primarily cultivated in Mediterranean environments, with additional acreage in North America and Asia (Martínez-Moreno et al., 2022; Mohammadi and Haghparast, 2022). Although it occupies approximately 5% of the global wheat area, it holds strategic importance for pasta production due to semolina color and strong gluten quality (Tidiane Sall et al., 2019; Webb, 2019; Ahmad et al., 2023), and cultivation continues to expand into Eastern Europe and Turkey (Beres et al., 2020; Xynias et al., 2020; Bożek et al., 2021). As climate change intensifies, increasing temperature variability and unseasonal frost pose a significant threat to durum wheat production both in traditional areas and in emerging regions. Late‐season frost during flowering induces spikelet sterility and grain abortion, causing severe yield losses often greater than in bread wheat (*T. aestivum* L.) (Fuller et al., 2007; Zheng et al., 2015; Beres et al., 2020). Although temperate-region farmers already favor spring‐sown varieties to avoid winterkill, increasingly mild winters encourage earlier sowing to exploit a longer season but heighten exposure to spring cold snaps, leading to substantial reproductive damage and yield loss (Collier et al., 2020).

Several studies on diverse durum wheat germplasm collections have revealed substantial genetic variation for key adaptive traits, including freezing tolerance (Longin et al., 2013; Göçmen Taşkın et al., 2020; Sieber et al., 2016). Encouragingly, some elite durum lines exhibit freezing tolerance comparable to bread wheat without compromising grain protein or gluten strength, indicating that freezing tolerance can be improved without detriment to high pasta-making quality (Longin et al., 2013; Göçmen Taşkın et al., 2020).

Modern durum wheat has a narrow genetic base due to historical domestication bottlenecks and intensive selection during the Green Revolution (Kabbaj et al., 2017; Maccaferri et al., 2019; Mazzucotelli et al., 2020; Soriano et al., 2021; Balla et al., 2022). Early genomic analyses indicate that cultivated durum retains only about 16% of the nucleotide diversity found in its wild ancestor, wild emmer (Haudry et al., 2007), and subsequent breeding further eroded allelic richness (Abu-Zaitoun et al., 2018). As a result, many alleles associated with stress tolerance and adaptive traits are rare or absent in current elite cultivars. Recent genomic surveys using high-density SNP arrays on diverse germplasm panels are now exposing this hidden variation, offering a largely untapped resource for breeding (Mengistu et al., 2016; Kabbaj et al., 2017; Taranto et al., 2020; Esposito et al., 2022; Gesesse et al., 2023).

Genome-wide association studies (GWAS) have served as a powerful tool in durum wheat breeding, enabling the dissection of complex traits related to abiotic stress tolerance and grain quality. For freezing tolerance a copy number variation at the *CBF-A14* gene within the *Fr-A2* locus on chromosome 5A, explains over 90% of the genotypic variance, making this locus a major breeding target for winter hardiness (Sieber et al., 2016). For grain quality, stable QTL for gluten strength have been mapped on chromosomes 1A and 1B, with associated markers proposed for marker-assisted selection to improve pasta-making quality (Johnson et al., 2019).

In durum wheat, as in other crops, the selection of parental lines is a critical decision that directly influences the success and efficiency of a breeding program. This step becomes particularly complex when multiple traits, often with significant trade-offs, must be improved simultaneously. To address this challenge, selection indices have become essential tools for identifying optimal parental selection tailored to specific breeding pipelines or target markets. One of the earliest and widely used selection indices is the Smith Index (Smith, 1936), which estimates the genetic merit of a line as a linear combination of trait breeding values weighted by their respective economic values. More recently, Crossa et al. (2025) showed that the Smith Index is effective for estimating net genetic merit, predicting selection response, and assessing its correlation with total genetic value. However, because assigning accurate economic weights is often tricky in practice, alternative approaches have been developed. Among these, the desired gain index (Pešek and Baker, 1969) offers a strategy for selection based on target goals rather than economic weighting.

In recent years, genomic selection (GS) has revolutionized plant breeding (Meuwissen et al., 2001; Lorenz et al., 2011). GS models integrate genotypic and phenotypic data to train predictive algorithms, which are then used to estimate the genomic estimated breeding values (GEBVs) of the target population based solely on their genotype data (Meuwissen et al., 2001). Applying GS in plant breeding programs will usually result in simultaneously enhancing the genetic progress rate and decreasing the breeding cycle time (Alemu et al., 2024; Escamilla et al., 2025). Building upon this framework, Ceron-Rojas et al. (2015) introduced the genomic selection index (GSI), demonstrating that GSI can outperform traditional phenotypic selection indices in terms of genetic gain per unit time. More recently, studies by Chung and Liao (2020, 2022) highlighted the benefits of integrating GEBV-based indices with algorithms that optimize genetic diversity. This combined approach has shown promise in identifying superior parental lines that balance immediate breeding goals with long-term genetic gains.

Therefore, in this study, GWAS was used to dissect the genetic basis of freezing tolerance and grain quality traits, while predictive modelling was applied for parental selection to optimize trait combinations. Specifically, our objectives were to: (i) characterize the genetic diversity and population structure of a durum wheat panel; (ii) identify MTAs associated with freezing tolerance and quality traits; (iii) develop genomic prediction models to optimize parental selection; and (iv) develop and validate KASP markers in the BC_2_F_2_ population.

## 2 Materials and methods

### 2.1 Plant materials and phenotyping

A panel of 250 durum wheat (*T. turgidum* L. subsp*. durum* (Desf.) Husn.) genotypes was used in this study, comprising 209 registered cultivars and 41 advanced breeding lines. Based on available breeder information and adaptation patterns, 67 genotypes were classified as winter type and 183 as spring type. The panel included germplasm commonly grown in Southern Europe (mainly Italy and France), as well as Central and Eastern Europe (e.g., Germany, Hungary, Russia). Detailed information on genotype origin, breeding background, and growth habit is provided in **Supplementary Table S3**.

Field trials were conducted under rainfed conditions over two consecutive growing seasons, 2019–2020 and 2020–2021, in two locations: at CREA Research Centre for Cereal and Industrial Crops (CREA-CI), Foggia, Southern Italy (41°27′ N, 15°30′ E, 70 m asl), and at Department of Breeding and Seed Production of Wheat and Triticale, National Grain Center P.P. Lukyanenko, Krasnodar, Russia (45°02′ N, 38°56′ E, 30 m asl).

In Italy, the genotypes were evaluated for grain quality using an augmented experimental design (Federer, 1956; Zhang et al., 2018) consisting of four blocks. All genotypes were tested in a single plot per season (i.e., unreplicated). To estimate spatial variation and experimental error, a widely cultivated check cultivar cv. Marco Aurelio, derived from Orobel//Arcobaleno/Svevo and released in Italy in 2010 was replicated four times per block, for a total of 16 check plots per season. Field trials in Italy were conducted under rainfed conditions, following standard agronomic practices commonly adopted by local farmers. Crops were sown at a conventional density of 350 seeds/m², with rows spaced 0.17 m apart. Fertilization included pre-sowing application of nitrogen (45 kg/ha) and phosphorus (115 kg/ha), followed by an additional nitrogen application (85 kg/ha) at the tillering stage. Weed, pest, and disease control were carried out using standard chemical treatments. Plots were harvested mechanically using a Wintersteiger Classic plot combine.

In Russia, freezing tolerance experiments were carried out under controlled conditions following the protocol described by several authors (Sutka, 1994; Fedoulov, 1997; Whaley et al., 2004; Wang et al., 2012; Li et al., 2015a; 2015b; 2015c). The experimental design consisted of a completely randomized design with two replications per genotype. After sowing 100 seeds per genotype per replication in 1×1 m soil boxes under natural field conditions until plants reached the 2–3 leaf stage (BBCH 12-13), seedlings were then hardened and then moved to a freezing chamber. They were kept at -5 °C for 24 hours to enhance hardening, followed by a gradual temperature decrease of 1 °C per hour until reaching -12 °C. Plants were maintained at this freezing temperature for 24 hours and then warmed up at 1 °C per hour to +5 °C. After an additional 24 hours at +5 °C, the boxes were transferred to a greenhouse supplied with Hoagland’s solution and grown in a growth chamber at 17 °C and 10 h/light (180 µE m^-2^ sec^-1^) and at 10 °C and 14 h/dark. The freezing tolerance (FT) was calculated as the number of green plants that survived 21 days after the freezing treatment (–12 °C), expressed as a percentage of the 100 seeds sown per genotype per replicate. Since the response variable was percentage-based, the data were transformed using the arcsine square root method prior to statistical analysis to stabilize variance.

Grain quality traits were evaluated only for the Italian trials. After mechanical harvest, grain samples were stored under refrigerated conditions until analysis. The traits were measured as follows: Thousand Kernel Weight (TKW) was determined on 500 seeds and expressed in grams. Wet Gluten Content (WG) and Gluten Index (GI) were measured using the ICC Standard Method No. 137/1 and 155; SDS Sedimentation Volume (ml), was evaluated according to the method of Pena et al. (1990); Test Weight (TW) was expressed in kg/hL and determined using the ICC Standard Method No. 081/1. All grain quality traits were determined in the first year (2019-20), while TKW was determined for two growing seasons.

### 2.2 Statistical analysis of phenotypic data

Because the Italian nursery in 2019–2020 and 2020–2021 was sown in a single, non-randomized layout with a repeated check genotype, we first controlled for spatial heterogeneity. For each test plot, we calculated the deviation of the nearest repeated-check plot from the overall mean of the check across the field and applied an additive correction:


$$y_{g,p}^{ad_{j}}=y_{g,p}-({\bar{C}}_{N(p)}-\bar{C})$$

where $y_{g,p}$ is the observed value of genotype g at plot p, ${\bar{C}}_{N(p)}$ is the mean of the check plots nearest to plot p, and $\;\bar{C}$ is the global mean of the check across the field.

For the four grain-quality traits (WG, GI, SDS sedimentation volume, and TW), only 2019–2020 data were available, and the same local check adjustment was applied to those single-year means. For the two agronomic traits (FT and TKW), adjusted mean values across the two growing seasons (2019–2020 and 2020–2021) were used. Phenotypic differences among genotypes were statistically assessed using the non-parametric test. All tests were performed in R (v4.4.0) using the *wilcox.test()* function and the ggpubr package (Kassambara, 2023).

For the GWAS analysis, best linear unbiased predictions (BLUPs) for FT and TKW across the two years were estimated using a linear mixed as follows:


$$y_{ij}=\;\mu +Year_{i}+g_{j}+\epsilon_{ij}$$

Where $y_{ij}$ is observed phenotype of genotype *j* in year *i*. μ is the overall mean, Year is the effect of year i considered as fixed, g is the random genotype effects, assumed g∼ $N(0,\;\sigma_{g}^{2})$, $\epsilon_{ij}$ is the random residual error, assumed $\epsilon_{ij}$∼ $N(0,{\text{ σ}}^{2})$. The fixed Year effect was formally tested in this model to confirm a year-to-year environmental influence on the traits. For the grain-quality traits, the single-year values were directly used as phenotypes.

### 2.3 Genotyping and population structure analysis

Genomic DNA was extracted from leaves by applying the CTAB method (Sharp et al., 1988). Genotyping was performed by Trait Genetics (Gatersleben, DE) using the Illumina ® iSelect 25K wheat SNP array (Muqaddasi, 2017). The physical positions of all SNPs were obtained by aligning sequences to the Svevo v1 reference genome (Maccaferri et al., 2019), retrieving only hits with a full-length alignment. Individuals with > 5% missing genotypes, SNPs with > 10% missing rate, and a minor allele frequency (MAF) < 0.05 were excluded. Population stratification and genetic structure analysis were performed using three different approaches: i) Principal Component Analysis (PCA), ii) Discriminant Analysis of Principal Components (DAPC), and iii) ADMIXTURE (Alexander et al., 2009). PCA and DAPC were carried out using the “adegenet” R package (Jombart, 2008). Before performing the structure analyses, SNPs were pruned at a linkage disequilibrium (LD) threshold of r² = 0.5 to reduce marker redundancy. The optimum number of clusters for DAPC was determined using the *find.clusters* function with 120 random starts (lowest Bayesian Information Criterion, BIC value). Cross-validation was used to identify the optimal number of principal components, based on the minimum of root mean square error in the repeated stratified sampling approach. ADMIXTURE v1.23 (Alexander et al., 2009; Alexander and Lange, 2011) was run for K = 1 to 20 using 5-fold cross-validation with 50 replicates.

Genomic regions potentially under selection were identified using the single-locus fixation index (Fst) values estimated by the Weir and Cockerham (1984) method using PLINK 1.9 (Chang et al., 2015). The Fst was computed based on PCA-defined groups. The loci above the $99^{th}$ percentile were considered potential outliers. The analysis of molecular variance (AMOVA) was performed with the poppr package (Kamvar et al., 2014), where Fst-like parameters (Φ) are estimated as a proportion of variance components that are derived from a pairwise matrix of squared Euclidean distances between individuals (Excoffier et al., 1992; Meirmans, 2006).

### 2.4 Genome-wide association study

Genome-wide association analysis for six evaluated traits was performed using the Fixed and Random Model Circulating Probability Unification (FarmCPU) method (Liu et al., 2016), implemented in the GAPIT3 package (Wang and Zhang, 2021) using R version 4.4.0 (R Core Team, 2024). Population structure was accounted for by including the first three principal components as covariates in the model. The significance of marker–trait associations (MTAs) was evaluated using two multiple testing procedures. First, a Bonferroni correction was applied using a threshold of p < 4.53 × 10^−6^ (equivalent to –log_10_(p) > 5.34). Second, a Benjamini–Hochberg (Benjamini and Hochberg, 1995) false discovery rate (FDR) adjustment was employed. Only SNPs passing both thresholds were considered statistically significant and retained for downstream interpretation and visualization. For visualization purposes, association strength was expressed as $LOD=-log_{10}(P)$ (hereafter referred to as LOD scores). Manhattan and quantile–quantile (QQ) plots were generated using custom R scripts with the ggplot2 (Wickham, 2016) and ggrepel (Slowikowski, 2024) R packages.

### 2.5 Predictive models for parental selection

Four standardized traits, FT, TKW, WG, and GI, were analyzed using a multi-trait genomic best linear unbiased prediction (GBLUP) model. The model also incorporated the genomic relationship matrix (VanRaden, 2008), computed from filtered molecular marker data, and was implemented using the BGLR package in R (Pérez-Rodríguez and De Los Campos, 2022). Genomic estimated breeding values (GEBVs) were calculated for each target trait based on this model, and were subsequently used as input in the parental selection framework proposed by Chung and Liao (2022), implemented through the IPLGP R package. Specifically, the function *simu.GEBVGD* was used, which integrates GEBVs with a genetic diversity algorithm to guide parental selection. To optimize performance across all four traits simultaneously, all selection directions were set to “inf” (i.e., favoring increased values). Additionally, a Genomic Selection Index (GSI) was applied using the following formula: $GSI=w_{FT}GEBV_{FT}+\ldots +w_{GI}GEBV_{GI}$. The trait weights reflected breeding priorities: 0.45 (FT), 0.05 (TKW), 0.25 (WG), and 0.25 (GI). Simulations were carried out up to the 8 generations (F8), and the mean GEBV per generation was recorded as a measure of genetic progress over time. Based on the combined GEBV-GD strategy and GSI ranking, a total of 40 potential parental lines were selected as the most promising candidates for multi-trait improvement and were subsequently considered for crossing and population development.

### 2.6 KASP marker development and validation on BC_2_F_2_ populations

From the 40 selected lines, six parents were chosen for crossing based on complementary trait profiles, genetic diversity representation, and seed availability; five donor x recurrent combinations were prioritized to generate BC_2_F_2_ populations (**Supplementary Table S4**). Specifically, cold-tolerant genotypes of Eastern European origin, namely Sinora and Yantarina were crossed to the high-quality Mediterranean cultivars Floridou, Fuego, Obelix, and Olimpo, which served as recurrent parents. Based on the prioritized parental combinations, five BC_2_F_2_ populations were developed from the following pedigrees: Olimpo/Sinora//Olimpo, Yantarina/Obelix//Obelix, Yantarina/Sy Lido//Sy Lido, Fuego/Sinora//Fuego, and Floridou/Sinora//Floridou. The crossing scheme followed a standard backcross protocol: F_1_ plants from the initial cross were backcrossed twice to the recurrent parent (BC_1_ and BC_2_), and the resulting BC_2_ plants were self-pollinated to generate BC_2_F_2_ populations (**Supplementary Table S5**). Each BC_2_F_2_ population consisted of approximately 50 individuals.

The BC_2_F_2_ populations were evaluated for freezing tolerance during the 2022–2023 growing season at the Department of Breeding and Seed Production of Wheat and Triticale, National Grain Center P.P. Lukyanenko, Krasnodar, Russia, following the same controlled freezing protocol described in section 2.1. Each BC_2_F_2_ population was tested using a completely randomized design with individual plants as experimental units, and their respective parents (recurrent and donor) as repeated checks. Surviving plants after the freezing treatment were recorded, and DNA was extracted from leaf for subsequent KASP screening. As for grain quality, due to the limited amount of grain available in these early generations, it was not possible to directly correlate the KASP results at this stage.

Three KASP assays were employed to screen all the lines (**Supplementary Table S5**). For freezing tolerance, the marker *RAC875_c101391_521* on chromosome 5A detected by the GWAS was selected. In addition, to validate the robustness of the new KASP assay, the marker *S1862541* (Würschum et al., 2017) was used as a positive control. For grain quality, the well-known marker *Glu-B1* (*Bx7OE_866*) located at 588 Mb was chosen. KASP assays were designed by LGC’s KASP-by-Design service. Each assay consisted of two allele-specific forward primers, one tailed with the standard FAM sequence, the other with the VIC sequence (FAM:5′-GAAGGTGACCAAGTTCATGCT-3′; VIC:5′-GAAGGTCGGAGTCAACGGATT-3′) and one common reverse primer, with the SNP positioned at the 3′ end of each allele-specific primer. KASP reactions were prepared by LGC in 5 μL volumes in 96-well plates. A typical 5 μL reaction contained ~45 ng dried DNA, 2.5 μL 1×KASP Master Mix (LGC Biosearch Technologies), and ~0.1 μL of a primer mix. PCR amplifications were run on a real-time thermal cycler using ABI ViiA7 (Applied Biosystems, Foster City, CA, United States) using a touchdown protocol as follows: initial denaturation at 94 °C for 15 min; 10 touchdown cycles of (94 °C 20 s, 61 °C→55 °C –0.6 °C per cycle, 60 s); then ~26 additional cycles of (94 °C 20 s, 55 °C 60 s). Fluorescence was read below 37 °C, and allelic discrimination plots were drawn using the SNP viewer software (https://www.biosearchtech.com/support/tools/genotyping-software/snpviewer).

To assess the association between the KASP markers and the phenotypic response, percentage survival was compared among genotype classes using the Kruskal–Wallis rank-sum test (Kruskal & Wallis, 1952). When the overall test was significant, pairwise genotype comparisons were performed using Dunn’s multiple-comparison test (Dunn, 1964) with Benjamini–Hochberg FDR adjustment (Benjamini and Hochberg, 1995) as implemented in the FSA R package (Ogle et al., 2023).

## 3 Results

### 3.1 Genetic diversity and population structure

**Figure 1:**
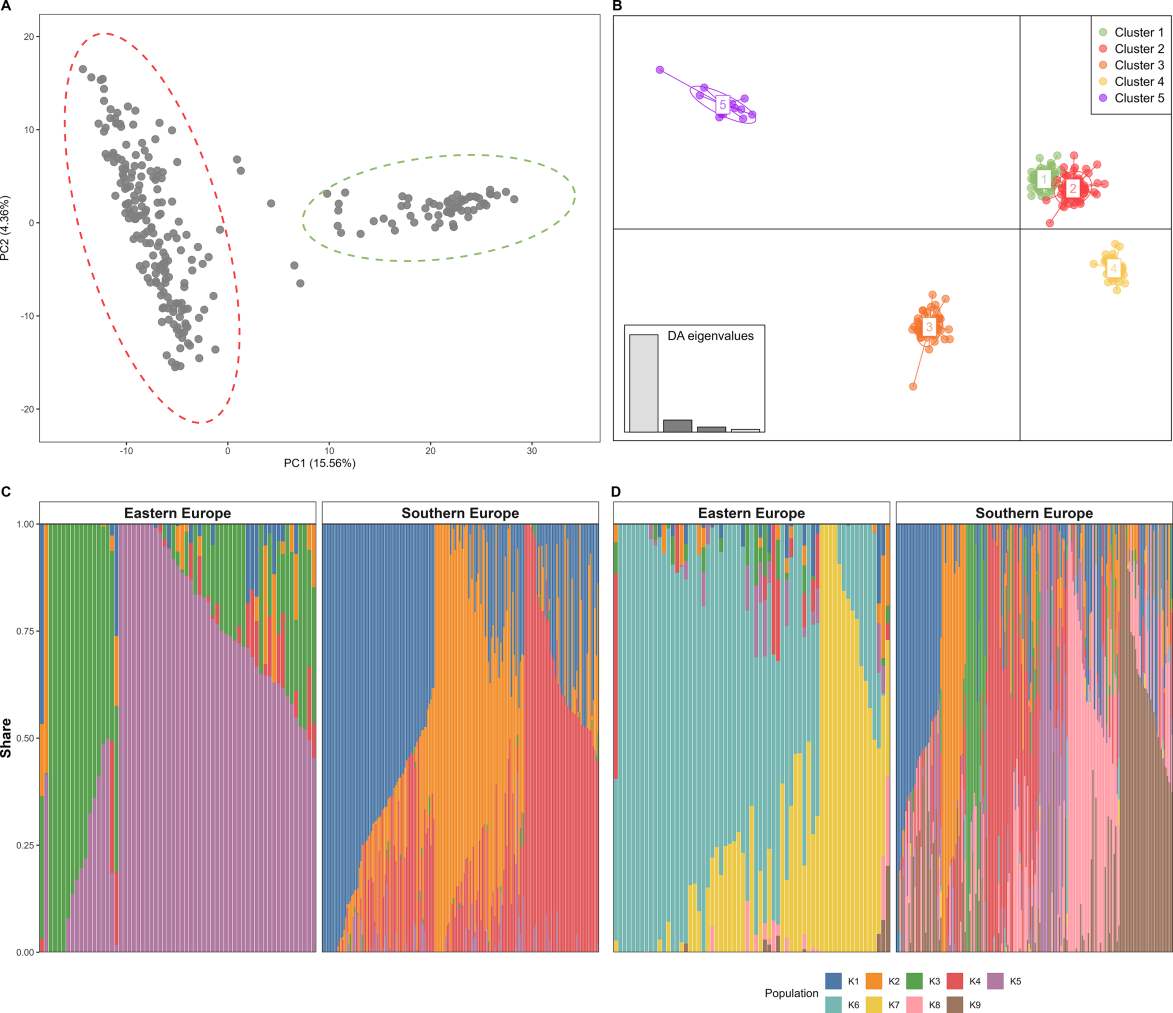
Population structure of 250 durum wheat accessions. **(A)** Principal Component Analysis (PCA) based on 6,409 SNP markers. The first two principal components (PCs) are shown. Each point represents one accession, whereas ellipses indicate geographic groups. **(B)** Discriminant analysis of principal components (DAPC) considering K = 5. Points represent individual accessions colored according to their cluster assignment. **(C)** Admixture-based ancestry fractions of individual accessions at K = 5. Accessions were ordered by their geographical origin. Each bar corresponds to a single accession colored according to membership coefficients in the inferred ancestral populations. **(D)** Admixture-based ancestry fractions of individual accessions at K = 9.

The scatter plot of the first (PC1) and second (PC2) components of PCA clearly distinguished the origin of accessions, allowing the classification in Southern European lines (distributed along PC2), and Eastern European accessions (PC1) (**Figure 1A**). Discriminant Analysis of Principal Components (DAPC) provided a more refined representation of the population structure within the studied panel (**Figure 1B**). K = 5 was identified as the optimal number of genetic clusters, which broadly reflects the pattern observed in the PCA (**Supplementary Figure S1A**; **Figure 1B**). The first three clusters (with 58, 70, and 58 individuals, respectively) were predominantly composed of accessions from the PCA-defined Group I (mainly Southern European), while Clusters IV and V (with 49 and 15 individuals) comprised genotypes from Eastern European (Group II). Clusters I and II were closely related, reflecting some degree of shared genetic backgrounds (**Figure 1B**). Similarly, Cluster IV showed a higher genetic affinity to the Southern European accessions, compared to the other Eastern European clusters. Admixture analysis further partitioned the panel into nine ancestral populations (K = 9), providing a finer resolution of genetic background within the panel. However, the overall structure tended to be homogeneous at both K = 5 and K = 9 (CV error decreased from 0.804 at K = 5 to 0.783 at K = 9; **Figures 1C, D**; **Supplementary Figure S1B**). At K = 5, groupings were fully consistent with the PCA and DAPC clusters, showing three subpopulations in Southern and two subpopulations in Eastern European varieties **Figure 1C**). Analysis of Molecular Variance (AMOVA) based on PCA and DAPC-defined groupings showed that 26.11% of the variation was attributable to the differences between the Southern and Eastern European groups, while 64.62% was explained by variation within accessions (**Supplementary Table S1**). All variance components were significant based on Monte Carlo permutation tests (9,999 iterations; P < 0.0001). The ΦST value of 0.354 indicated substantial overall genetic differentiation, and ΦOrigin-total (0.261) quantified the divergence between PCA-defined groups, supporting the population structure inferred from PCA and DAPC. As shown in **Supplementary Figure S2**, single-locus Fst analysis on PCA identified groups revealed a total of 62 SNPs that exceeded the 99th percentile threshold. These SNPs were distributed across several chromosomes, with most outliers on chromosome 5A (n = 34) followed by chromosomes 5B (n = 12) and 6B (n = 6), and fewer chromosomes 1A, 1B, 2A, 3B, 6A, and 7A. These loci likely reflect regions affected by divergent selection or region-specific breeding between the two populations.

### 3.2 Phenotypic variation in field trials

**Supplementary Table S2** provides an overview of the descriptive statistics for agronomic and quality traits. Freezing tolerance at -12 °C (FT) showed high variability (CV > 120%) over both years due to the presence of a large number of susceptible genotypes exhibiting complete mortality under freezing conditions (-12 °C). In total, 108 genotypes in 2020 and 121 genotypes in 2021 exhibited complete mortality (0% survival), while 84 genotypes (33.6%) were entirely susceptible across both years, indicating considerable genetic vulnerability to freezing stress within the panel. No genotype demonstrated consistent complete resistance (100% survival) in both years; however, eight genotypes achieved full survival in 2021, suggesting a possible influence of year-specific environmental conditions on freezing tolerance. Consistent with this overall year-to-year variability, a linear mixed model detected a significant Year effect on freezing survival (F = 12.69, P = 0.00044), with the mean survival increasing from 18.8% in 2020 to 24.6% in 2021. Genotype rankings across the two seasons were only moderately consistent (Spearman’s ρ = 0.666, P < 2.2 × 10^−16^), supporting the view that no genotype maintained fully stable high resistance. Although Southern European genotypes were predominantly susceptible, a small subset exhibited moderate freezing survival, with five accessions Obelix, Olimpo, Tripudio, Floridou, and Nautilur, showing two-year mean survival rates above 40% (range: 43.4–69.8%), indicating the presence of limited but detectable freezing tolerance within Southern germplasm. Thousand Kernel Weight (TKW) showed lower variations, and the pattern of productivity and variation was consistent among the years (Mean=39.5 & 41.6; CV = 10.5 & 10.7). Among the quality traits, Wet Gluten (WG), Gluten Index (GI), and SDS sedimentation Volume Test (SDS) showed a considerable variation (CV ≈ 25–27%), while the evaluated genotypes panel exhibited a low variation in Test weight (TW) (CV = 2.1%), an indicator of grain bulk density.

The phenotypic comparisons of the studied traits among the genetic groups defined by PCA, DAPC (five groups) and ADMIXTURE (K=5) are summarized in **Table 1**. For comparison based on Admixture clusters, individuals were assigned to five groups (K1–K5) according to their highest Q-matrix membership coefficient. The phenotypic comparison of the PCA-defined groups is shown in **Supplementary Figure S3**. FT showed a highly significant difference between groups (p < 2.2 × 10^−16^), with substantially higher survival observed in Eastern European genotypes compared to those from Southern Europe. Notably, 83 out of 181 Southern European accessions (≈46%) were completely susceptible to freezing (0% survival), whereas none of the Eastern European genotypes exhibited complete mortality under the same conditions. Southern European accessions exhibited higher average and variation in TKW. The quality traits, except for TW, were significantly different between groups at a very high level (p < 1.3 × 10^−11^). Southern European genotypes possessed consistently high WG contents, whereas Eastern European lines were more variable, with some accessions exceeding WG Southern counterparts. The differences were more pronounced for SDS and, particularly, GI, both of which were significantly higher in Southern European genotypes. Test Weight showed almost similar performance level and pattern of variation among the groups. Among Eastern European genotypes, the best-performing entries for grain quality under Italian field conditions included DF-00091, Koshelevskaja, MVTD12-99, Blindur/DF38-86/3, and Condur.

**Table 1:** Performance evaluation of traits across genetic groups defined by PCA, DAPC, and ADMIXTURE.

Method	Group	n	FT (%)	TKW (g)	TW (kg/hl)	GI (%)	WG (%)	SDS (ml)
PCA	Eastern Europe	64	61.1 ± 16.51	39.01 ± 2.36	82.2 ± 1.58	54.81 ± 16.69	39.68 ± 11.96	38.94 ± 14.41
	Southern Europe	181	7.33 ± 12.14	41.21 ± 3.78	82.23 ± 1.81	94.48 ± 12.1	30.35 ± 5.06	54.96 ± 8.46
DAPC	Cluster I	58	8.04 ± 12.94	42.34 ± 4.05	81.82 ± 2.02	93.08 ± 16.95	30.17 ± 5.82	53.14 ± 8.85
	Cluster II	70	4.76 ± 10.44	40.89 ± 3.97	83.01 ± 1.64	94.85 ± 9.39	30.35 ± 8.5	53.63 ± 8.44
	Cluster III	58	12.25 ± 16.45	40 ± 3.47	81.76 ± 1.45	93.68 ± 11.77	32.29 ± 4.51	56.97 ± 9.03
	Cluster IV	49	59.5 ± 17.32	39.13 ± 2.42	82.26 ± 1.72	50.24 ± 14.13	42.71 ± 11.48	36.09 ± 15.03
	Cluster V	15	66.35 ± 12.64	38.63 ± 2.2	82 ± 1.05	69.74 ± 16.03	29.81 ± 7.42	48.27 ± 6.23
ADMIXTURE	K1	76	4.48 ± 10.07	40.83 ± 3.93	82.92 ± 1.65	94.82 ± 9.54	30.63 ± 8.28	53.74 ± 8.76
	K2	60	12.78 ± 16.67	40.01 ± 3.42	81.74 ± 1.47	93.9 ± 11.62	32.14 ± 4.62	57.13 ± 8.93
	K3	18	67.73 ± 12.52	38.89 ± 2.3	82.01 ± 1.17	65.77 ± 17.19	31.08 ± 7.47	47.47 ± 8.28
	K4	49	6.87 ± 9.69	42.67 ± 4.13	81.8 ± 2.06	93.65 ± 16.5	29.57 ± 5.69	53.1 ± 7.53
	K5	47	58.68 ± 17.11	39.15 ± 2.45	82.31 ± 1.73	50.38 ± 14.42	43.03 ± 11.62	35.41 ± 14.88

FT, Freezing tolerance at -12^o^C; TKW, Thousand kernel weight; TW, Test weight; GI, Gluten index; WG, Wet gluten; SDS, SDS sedimentation volume test.

DAPC clusters IV and V, being highly represented by Eastern European genotypes, revealed the highest FT (59.50 ± 17.32% and 66.35 ± 12.64%, respectively), which corresponds to the cold-tolerant trends in PCA-defined Eastern groups. Similarly, admixture clusters K3 and K5, enriched mainly for Eastern European ancestry, also displayed similar high freezing tolerance rates (67.73 ± 12.52% and 58.68 ± 17.11%).

For quality traits, Admixture groups K1, K2, and K4 and DAPC groups I-III showed high GI (≥ 93) and SDS (> 53 ml). However, the trend for WG content was more complicated: while DAPC clusters I-III and Admixture K1, K2 & K4 had high consistent average values (~30–32%), extremely high WG percentages were also present in DAPC cluster IV (42.71 ± 11.48%) and Admixture group K5 (43.03 ± 11.62%), both of which were largely comprised of Eastern germplasm. On the other hand, DAPC cluster V and Admixture group K3 also mainly composed of Eastern accessions, showed comparatively moderate WG levels, similar to or even lower than those observed in Southern clusters. Test weight showed constant performance and did not differ appreciably among clusters identified by either method.

### 3.3 GWAS for agronomic and grain quality traits

**Figure 2:**
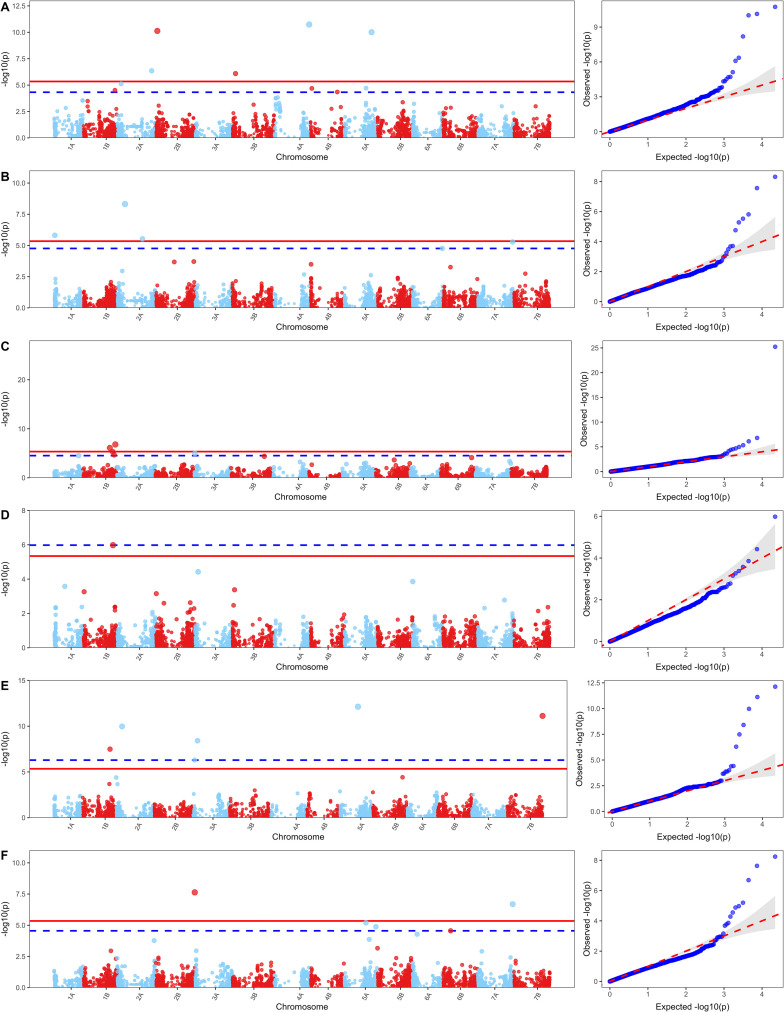
Genome-wide association scans for six agronomic and grain-quality traits. For each trait, the Manhattan plot is shown on the left and the corresponding Q–Q plot on the right. **(A)** Freezing tolerance, **(B)** TKW, **(C)** Wet Gluten Percent, **(D)** Gluten Index, **(E)** SDS test ml, **(F)** Hectoliter weight. The solid red line marks the Bonferroni threshold (experiment-wise α = 0.05; −log_10_ P ≈ 5.34 for 11,047 tests), while the blue dashed line shows the Benjamini–Hochberg FDR = 0.05 threshold.

A total of 23 significant MTAs were identified through GWAS, of which four fell in unmapped or non-consensus chromosomal regions and were therefore excluded. Five MTAs were significantly associated with FT and spanned chromosomes 2A, 2B, 3B, 4A, and 5A (**Table 2**; **Figure 2A**). *QFt-5A* on chromosome 5A had the highest effect (β = 6.01) and explained 27.08% of the phenotypic variance (PVE). Similarly, *QFt-2A* at 691Mb on chromosome 2A also showed a considerable effect (β =3.76) and explained 12.16% of the PVE. The remaining associated loci on chromosomes 2B (*QFt-2B*), 3B (*QFt-3B*), and 4A (*QFt-4A*) accounted for 6.4 to 11.3% of the variation (**Table 2**). Three MTAs were detected for TKW, of which one was located on chromosome 1A (*QTkw-1A*) and two on chromosome 2A (*QTkw-2A.1* and *QTkw-2A.2*) (**Figure 2B**). *QTkw-2A.1* had the largest PVE (39.8%), whereas the other two markers explained 5.5% and 15.0% of PVE. Two MTAs on chromosome 1B (*QWg-1B.1* and *QWg-1B.2*) with low impact (PVE of 0.3% and 2.5%) were associated with WG (**Figure 2C**), whereas the *QGi-1B* at 595 Mb on chromosome 1B was significantly associated with GI (**Table 2**; **Figure 2D**). Six MTAs spanning chromosomes 1B, 2A, 3A, 5A, and 7B were found for SDS (**Figure 2E**). *QSds-3A.2* at 95 Mb on chromosome 3A and *QSds-5A* on chromosome 5A explained the highest PVE (30.2% and 24.2%, respectively), whereas the others showed low impact (PVE between 2.1 and 12.8%). Two MTAs for TW were detected, represented by *QTw-7A* on chromosome 7A and *QTw-2B* on chromosome 2B (**Table 2**; **Figure 2F**), which explained 35.6% and 21.6% of the phenotypic variance, respectively. To further qualify the GWAS results, phenotypic variation across genotype classes was assessed for all significant MTAs. Fifteen of the 19 significant MTAs showed significant differences based on Wilcoxon rank-sum tests (α = 0.05; **Supplementary Figure S4**), confirming the robustness of the detected associations. In addition, a favorable haplotype based on three MTAs strongly associated with freezing tolerance (higher or lower freezing tolerance) was observed mainly within Eastern European accessions (Groups IV and V from DAPC), whereas lines from the South had zero or one resistant allele, with no lines carrying resistant alleles at all three selected loci (**Supplementary Figure S5A**). In contrast, grain quality-associated favorable alleles were mainly distributed in Southern European groups (I-III) as single-allele carriers, with no Southern group lacking favorable alleles entirely, whereas Eastern European accessions differed markedly, with Group IV enriched for two favorable alleles and many lines in Group V not carrying any favorable allele (**Supplementary Figure S5B**).

**Table 2:** List of significant MTAs detected by FarmCPU.

QTL name	SNP	Chr	Position	LOD	MAF	Effect	PVE (%)
*QFt-2A*	CAP12_rep_c6956_169	2A	691749927	6.35	0.08	-3.76	12.16
*QFt-2B*	RAC875_c17197_504	2B	29946175	10.1	0.16	4.85	11.31
*QFt-3B*	AX-94440104	3B	65741446	6.09	0.05	4.58	9.18
*QFt-4A*	JD_c581_466	4A	707180653	10.7	0.30	3.34	6.48
*QFt-5A*	RAC875_c101391_521	5A	488243409	10.0	0.28	5.83	27.08
*QTkw-1A*	AX-94503555	1A	13945581	5.81	0.25	-0.58	5.49
*QTkw-2A.1*	BS00089457_51	2A	155892551	8.32	0.12	-1.28	39.75
*QTkw-2A.2*	AX-94436530	2A	505957410	5.53	0.09	-0.88	14.99
*QWg-1B.1*	RAC875_rep_c109215_398	1B	541156609	6.11	0.31	-1.91	0.318
*QWg-1B.2*	BobWhite_c9091_160	1B	652138386	6.80	0.15	4.16	2.47
*QGi-1B*	GENE-0506_129	1B	595944640	5.53	0.07	-7.54	1.497
*QSds-1B*	Tdurum_contig42852_667	1B	558563813	7.49	0.38	-2.88	2.1
*QSds-2A*	Kukri_c44442_274	2A	122727154	9.97	0.10	-6.31	12.82
*QSds-3A.1*	AX-94662400	3A	38406613	6.29	0.26	-2.81	2.82
*QSds-3A.2*	AX-94686079	3A	95695069	8.41	0.09	5.94	30.16
*QSds-5A*	AX-95216226	5A	368652154	12.1	0.16	7.65	24.18
*QSds-7B*	AX-94767893	7B	716676882	11.1	0.26	3.79	3.98
*QTw-2B*	tplb0042o21_419	2B	781593041	7.63	0.37	-0.44	21.64
*QTw-7A*	wsnp_Ku_c5693_10079343	7A	701939791	6.69	0.34	-0.38	35.57

Chr, Chromosome; MAF, Minor allele Frequency; LOD, Logarithm of Odds; PVE (%), Percentage of total phenotypic variance explained.

### 3.4 Simulated genomic selection for multi-trait improvement

**Figure 3:**
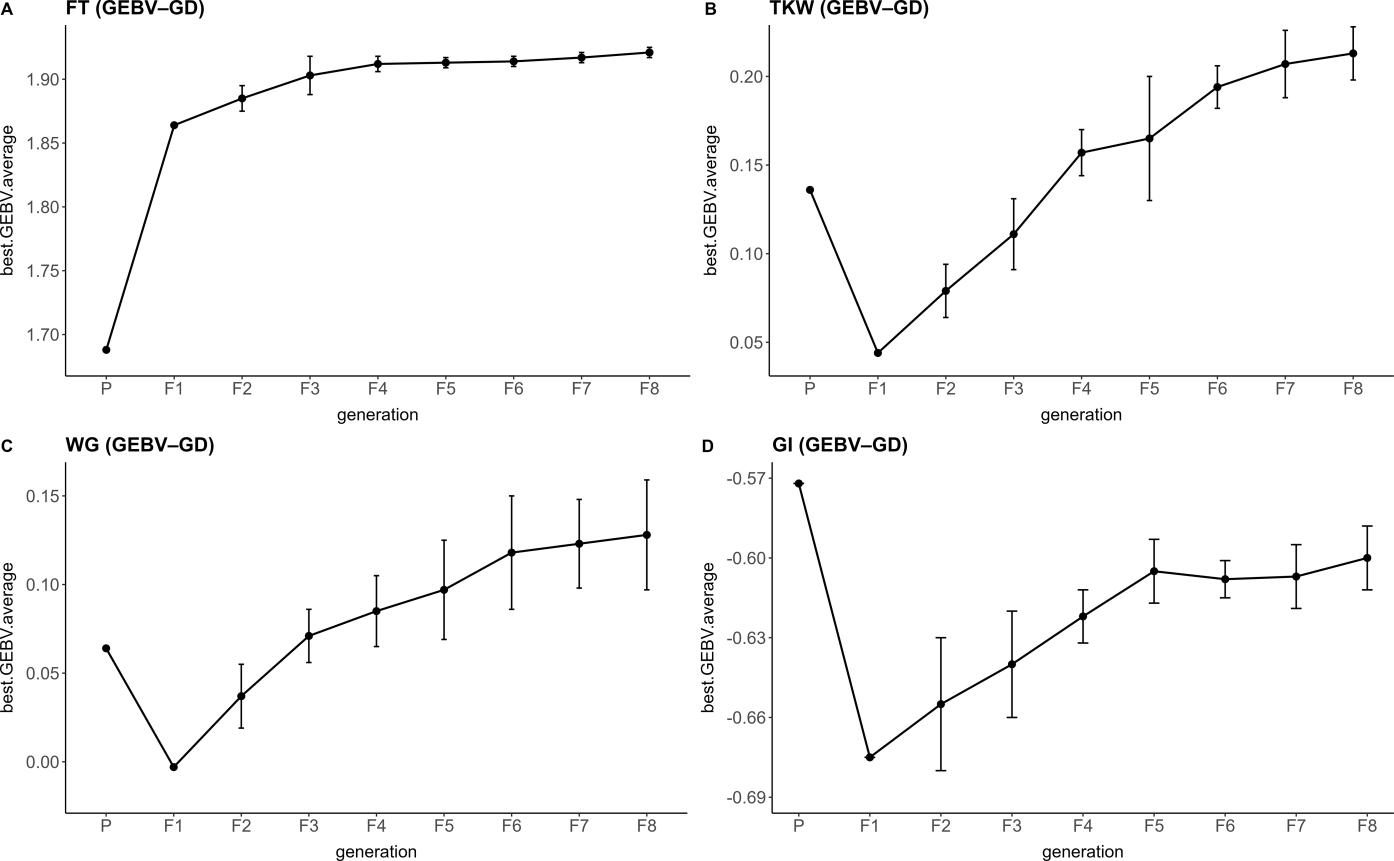
GEBV trends across generations under the GEBV-GD strategy. Line plots showing the progression of best-performing individuals’ average GEBVs from parental (P) to F8 generation for four traits: **(A)** Freezing tolerance **(B)** thousand kernel weight (TKW), **(C)** Wet Gluten (WG), and **(D)** gluten index (GI). Parental lines were selected using a multi-trait selection index that balances GEBVs and genomic diversity. GEBV averages were derived from simulated progeny populations. Error bars represent standard deviations across replicates.

A total of 40 parental lines were selected using a multi-trait genomic selection algorithm aimed at improving four target traits simultaneously (**Supplementary Table S4**). The simulation results were generally promising except for GI, showing consistent improvement in average GEBVs over generations (**Figure 3**). Notably, FT, which was assigned the highest selection weight (0.45), exhibited a marked increase in performance, where the GEBV rose steadily from approximately 1.70 in the parental generation (P) to nearly 1.90 in the F8 generation. Interestingly, although TKW was considered a secondary trait in the selection strategy, it demonstrated a strong positive trend, with average GEBV values increasing from around 0.05 in the parental generation to approximately 0.20 in F8. Additionally, WG also showed modest improvement, with GEBVs increasing from approximately 0.00 to slightly above 0.10 over the same period. In contrast, GI displayed a declining trend, with average GEBVs decreasing from –0.57 to –0.69 (**Figure 3**). Based on the genomic selection results, six parental lines namely Olimpo, Fuego, Sinora, SY Lido, Floridou, Yantarina, and Obelix, were used to develop five BC_2_F_2_ populations. The criteria for parental selection and the population development scheme are described in the Materials and Methods, and the resulting populations are reported in **Supplementary Table S5**.

### 3.5 Experimental validation in BC_2_F_2_ breeding populations

**Figure 4:**
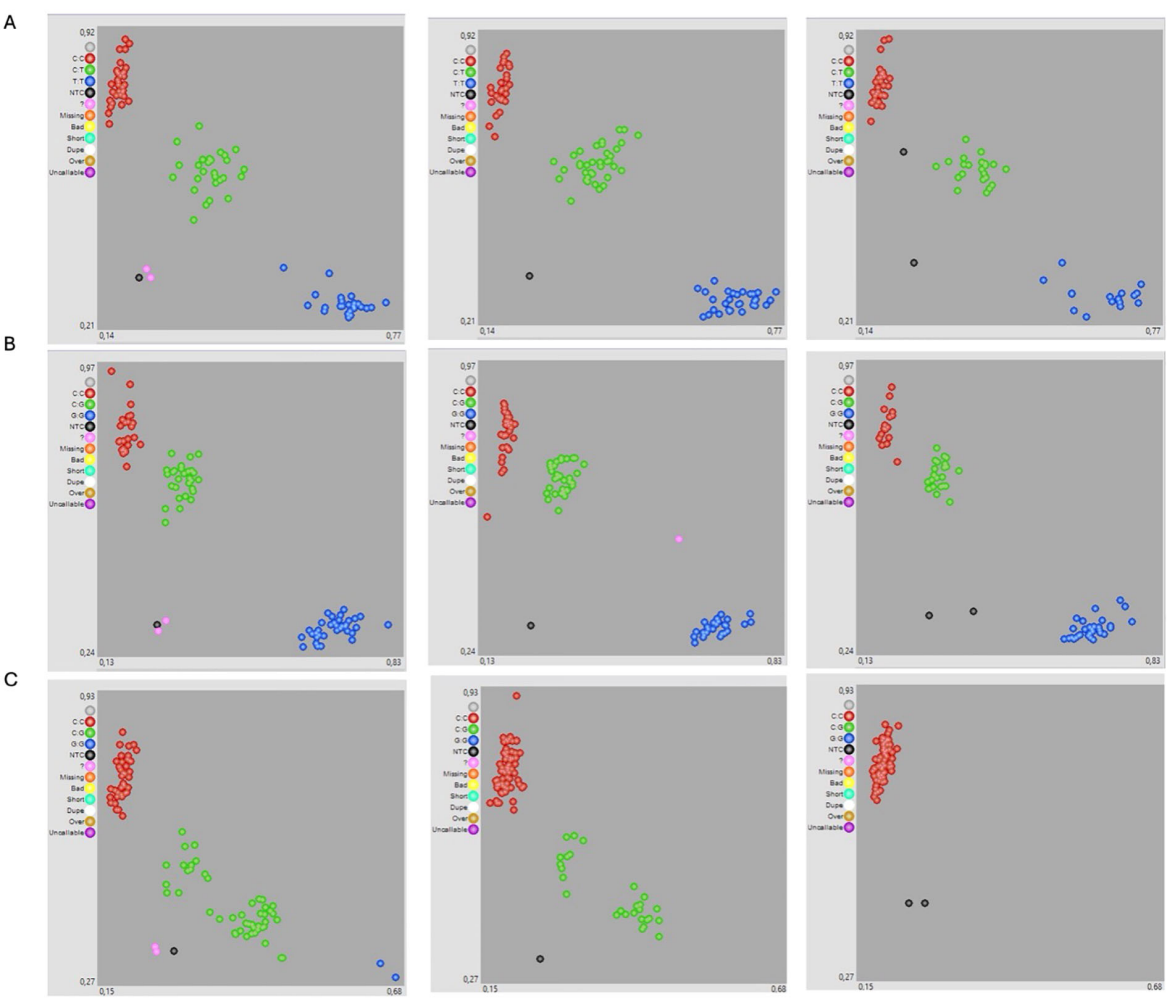
Scatter plot for three KASP assays in three F_2_BC_2_ populations developed following the parent selection approach. RAC875_c101391_521 **(A)** and S1862541 **(B)** were used for freezing tolerance, whereas *Glu-B1* (Bx7OE_866) **(C)** was used for quality. Red individuals have the HEX-type allele; blue individuals have the FAM-type allele. In both cases, individuals are homozygous for the reference or alternate allele. Green individuals are heterozygous for the allele. Black dots represent the negative control, and pink dots represent uncallable genotypes.

Among the markers used to screen the BC_2_F_2_ breeding populations, particular attention was given to the marker RAC875_c101391_521 (*QFT-5A*), which was identified in this study. This marker demonstrated strong reliability, showing clear genotype clustering in KASP assays (**Figure 4**) and consistent results with the positive control S1862541 (**Supplementary Table S5**). Statistically significant differences in FT were observed across genotype classes for both markers. For S1862541 SNP, the Kruskal–Wallis test yielded χ² = 54.31 (df = 2, p = 1.61 × 10^−12^), while it was χ² = 55.81 (df = 2, p = 7.62 × 10^−13^) for RAC875_c101391_521 SNP. *Post hoc* Dunn’s tests with Benjamini–Hochberg correction indicated that homozygotes for favorable alleles consistently outperformed heterozygotes and unfavorable homozygotes (**Supplementary Figure S6**).

For the *Glu-B1(Bx7OE_866)* marker associated with gluten strength, KASP genotyping (**Figure 4**) showed that 69.3% of lines carried the strong allele, 29.1% were heterozygous, and only 0.8% carried the weak allele (**Supplementary Table S5**). Notably, 80.3% of the freezing-tolerant lines also carried the strong-gluten allele, suggesting a promising combination of cold tolerance and end-use quality traits. Across the BC_2_F_2_ populations, around 60 lines were identified as carrying favorable alleles at both key loci (freezing tolerance and gluten quality), supporting the effectiveness of the parental selection approach and the relevance of the predictive model.

## 4 Discussion

### 4.1 Implications of genetic diversity analysis

The consistent population structure revealed by PCA, DAPC, and ADMIXTURE highlights the strong role of geographic origin and breeding history in shaping the genetic diversity of the durum wheat panel (**Figure 1**). Rather than reflecting purely neutral stratification, the observed separation between Eastern and Southern European accessions appears to be functionally linked to historical selection pressures and adaptive trajectories, probably reflecting contrasting agro-climatic conditions and breeding objectives. Eastern European accessions, originating from regions characterized by harsher winter conditions, showed a more uniform genetic background and consistently higher freezing tolerance. This pattern suggests long-term directional selection for cold resilience, which has contributed both to population differentiation and to the uneven distribution of freezing tolerance across clusters. In contrast, Southern European lines displayed higher levels of admixture and greater dispersion along principal components, consistent with extensive historical gene flow and targeted introgressions aimed at improving quality-related traits. These patterns are in line with Mediterranean breeding strategies that rely on repeated germplasm exchange and recombination to enhance pasta-making quality, rather than strict adaptation to extreme winter conditions.

Despite the clear separation between Eastern and Southern European accessions, the presence of accessions from Eastern Europe genetically closer to Southern European groups further supports the occurrence of historical connectivity and shared founder lines. These connections likely arose during post–Green Revolution breeding efforts, when quality-improving germplasm was widely exchanged across Europe, leading to partial convergence between otherwise distinct gene pools (Vavilov, 1951; Vavilov and Dorofeev, 1992).

Importantly, AMOVA indicated that most genetic variation resides among individual accessions rather than between predefined subpopulations (**Supplementary Table S1**). This pattern suggests substantial exploitable diversity within geographic groups, enabling effective parental selection and multi-trait improvement without relying exclusively on highly divergent germplasm. The identification of differentiated genomic regions on chromosomes 5A, 5B, and 6B known in the literature suggested that population structure may be shaped not only by demographic history but also by the selection of loci involved in cold tolerance, environmental adaptation, and grain quality (Taranto et al., 2020; 2023). Together, these findings indicate that the genetic structure of the panel is both historically and functionally meaningful, reflecting the joint effects of adaptation, breeding practices and selection for key agronomic traits.

### 4.2 Phenotypic variation and trait differentiation across genetic clusters.

Significant phenotypic divergence occurred between the two main genetic groups, reflecting contrasting adaptive histories and breeding priorities (**Supplementary Figure S3**). Eastern European accessions consistently showed superior freezing tolerance under −12 °C, with several genotypes maintaining high survival rates across years, whereas a substantial proportion of Southern accessions were highly susceptible, in line with earlier reports (Nazco et al., 2012; Soriano et al., 2021). The concordance between phenotypic performance and genetic classification across PCA, DAPC and ADMIXTURE indicates that freezing tolerance is not randomly distributed within the panel but is tightly associated with population structure shaped by long-term regional adaptation. The superior performance of Eastern accessions likely reflects long-term fixation of key freezing tolerance alleles such as *Fr-A2* and *Vrn-A1* (Whiting et al., 2025).

Conversely, Southern accessions outperformed their Eastern counterparts in quality-related traits, particularly for GI and SDS traits, consistent with breeding strategies prioritizing end-use quality over cold tolerance in Mediterranean environments (De Vita et al., 2007; Subira et al., 2014). Notably, certain Eastern accessions exhibited high WG content, indicating the presence of useful alleles that can be decoupled from gluten strength. The genetic proximity of these accessions to high-quality genotypes from Southern indicates that historical gene flow or shared founder lines may have contributed to quality-related variation within Eastern germplasm, thus these accessions represent a valuable resource for breeding strategies aimed at decoupling protein content from gluten strength, helping to overcome negative correlations that often constrain simultaneous improvement of yield, stress tolerance and end-use quality (Vida and Ottò, 2014a; Sieber et al., 2016). In the present study, this trend toward quality improvement was particularly evident in the most recent breeding lines developed in Eastern Europe, which showed a stronger genetic affinity with Southern European germplasm.

### 4.3 GWAS for agronomic and quality traits

GWAS identified 19 significant (MTAs) associated with six traits, including *QFt-5A* for freezing tolerance on chromosome 5A at 488 Mb, explaining over 27% of phenotypic variance (**Figure 2**; **Table 2**). The MTA co-localizes with the well-characterized *Fr-A2* region, known to harbor C-repeat binding factor (*CBF*) cluster genes central to cold acclimation (Babben et al., 2018). Copy-number variation of the *CBF-A14* gene in this region explained up to 90% of freezing tolerance variations in previous durum wheat studies (Sieber et al., 2016), confirming its value as a major target for freezing-tolerant lines. Additional loci with moderate effects (6–12% PVE) were identified on chromosomes 2A (*QFt-2A* at 691 Mb), 2B (*QFt-2B* at 29 Mb), 3B (*QFt-3B* at 65 Mb), and 4A (*QFt-4A* at 707 Mb). All these loci have been previously reported in the literature. Notably, *QFt-2A* colocalized with QTLs associated with kernels per plant (Peng et al., 2003), spikes per plant, and leaf number (Giunta et al., 2018). *QFt-2B* overlapped with genomic regions linked to heading date (Maccaferri et al., 2014), flag leaf appearance (Giunta et al., 2018), and grain yield (Milner et al., 2016). Similarly, *QFt-4A* corresponds to a well-characterized region: Gadaleta et al. (2011) reported a significant association with grain protein content, while Graziani et al. (2014) associated the same region with spikes per m². Collectively, these traits are known to be indirectly influenced by cold stress, supporting the relevance of the identified loci. For grain-quality traits, three MTAs on 1B spanning 541 Mb (*QWg-1B.1*) – 652 Mb (*QWg-1B.2*) were associated with WG and GI (**Table 2**). Their distinct effects suggest that protein content and gluten strength are genetically separable, a finding corroborated by long-term breeding progress in Italian and Spanish cultivars (Subira et al., 2014). The *Glu-B3* on 1BS, encoding low-molecular-weight glutenin subunits, likely underlies the observed effects. Chromosome 1B has long been recognized as a hotspot for gluten strength genes in durum wheat (Johnson et al., 2019), and allelic variation at these loci has profound effects on dough properties. Additional MTAs on chromosomes 1B (*QSds-1B*), 2A (*QSds-2A*), 3A (*QSds-3A.1* and *QSds-3A.2*), 5A (*QSds-5A*), and 7B (*QSds-7B*) were associated with SDS, broadening the set of markers that can guide selection for end-use quality. Among them, *QSds-2A* was also described by Fiedler et al. (2017) as associated with gluten strength, whereas *QSds-1B* was also described by Kidane et al. (2017) and Mengistu et al. (2016) as associated with heading date and days to maturity, suggesting an indirect relationship between phenology and SDS mediated by grain filling dynamics and environmental conditions.

Despite recent advances in mapping genomic regions and marker–trait associations underlying gluten strength and SDS sedimentation volume, improving gluten quality through breeding remains challenging due to its complex, quantitative, and environmentally responsive genetic architecture (Ruan et al., 2020) that limits the selection of a single marker (Johnson et al., 2019). By contrast, genomic selection offers a more powerful framework by integrating the combined effects of multiple loci and their interactions (Meuwissen et al., 2001; Battenfield et al., 2016).

### 4.4 Predictive breeding as a useful strategy for optimizing trait selection

Given the limitations of traditional MAS for complex quality traits, GS emerges as a promising alternative that can effectively capture the polygenic nature of key agronomic characteristics. In this study, a multi-trait GS model that incorporated genetic diversity proved effective in achieving simultaneous genetic gain across four targeted traits. By assigning the highest selection weight to FT, the model emphasized alleles associated with cold adaptation, and the steady improvement in FT across simulated generations demonstrates that this strategy can effectively translate selection pressure into genetic progress (**Figure 3**).

Wet gluten content and gluten index, which also received substantial weights, exhibited divergent trajectories. While WG showed moderate improvement, GI declined slightly over generations (**Figure 3**). This inverse trend suggests an underlying genetic trade-off between cold tolerance and certain aspects of protein functional quality, an antagonistic correlation well documented in wheat (Vida et al., 2014b; Kroupina et al., 2023; Dumlu et al., 2025). To mitigate such trade-offs in real breeding programs, restriction selection indices could be implemented (Mancin et al., 2022); this approach allows improvement of a target trait while constraining change in another, thereby preventing the deterioration of key quality attributes. These results highlight the importance of dynamically calibrating selection indices so that stress-adaptation gains do not erode end-use quality. Interestingly, TKW, which received a relatively low selection weight, improved consistently across generations. This likely reflects favorable pleiotropy or linkage with other prioritized traits, confirming that indirect selection can enhance secondary traits when genomic relationships are efficiently exploited (Ceron-Rojas et al., 2015). Overall, the GS-based indices provide a powerful framework to navigate complex trade-offs and improve multiple traits simultaneously. Nonetheless, the observed decline in GI highlights the need for careful monitoring of correlated responses and, when necessary, the use of trait constraints or dynamic weighting schemes to prevent erosion of key quality attributes while enhancing stress resilience and yield. These findings validate the utility of GS-based selection indices as a forward-looking tool in durum wheat breeding pipelines.

### 4.5 Breeding validation

The successful application of KASP assays for marker-assisted selection in BC_2_F_2_ populations confirmed the practical utility of GWAS-identified MTAs in early-generation selection (**Figure 4**). The validated markers, targeting major loci associated with freezing tolerance and gluten strength, enabled high throughput genotyping of segregating progenies and facilitated the early elimination of plants lacking favorable alleles. This is particularly advantageous in winter × spring crosses, where selection pressure must be applied rapidly and precisely to maintain both cold adaptation and end-use quality. *QFt-5A* on chromosome 5A, co-localizing with the *Fr-A2*, proved effective in enriching freezing-tolerant genotypes within progeny lines, consistent with results in bread wheat where KASP-based selection for *Fr-A2* significantly increased freezing tolerance in segregating populations (Michel et al., 2019; Song et al., 2023). This is noteworthy since KASP technology is widely adopted in bread wheat, but its deployment in durum wheat remains limited, particularly for traits like abiotic stress tolerance, which are historically under-represented in quality-oriented breeding programs (Natoli et al., 2021). Thus, our study contributes to filling this gap by providing a new KASP marker that can be successfully used in durum germplasm for key adaptive traits, in line with other studies (Esposito et al., 2024). Moreover, the combination with the well-known *Glu-B1* marker facilitated the effective selection of high-quality lines without compromising genetic gains in freezing tolerance. In our study, GWAS identified significant MTAs for grain quality traits co-localizing with *Glu-B1*. The concordance between our GWAS results and the well-established functional marker justified the adoption of the *Glu-B1* marker, rather than the development of a novel locus-specific marker. Although our grain quality phenotyping was conducted in one growing season, the detection of significant associations nearby *Glu-B1* validated by multiple independent studies, supports the robustness of this marker for selection purposes. This also confirms the feasibility of pyramiding traits with divergent physiological and genetic architectures using integrated genomic tools. Overall, our results emphasize that a well-designed breeding pipeline combining GWAS, predictive selection, and functional markers can significantly increase selection efficiency and shorten breeding cycles, especially for complex traits under dual selection pressures (e.g., climate resilience and product quality).

## 5 Conclusion

This study presents an integrated framework for accelerating the development of durum wheat germplasm combining enhanced freezing tolerance and pasta-making quality. Comprehensive genotypic and phenotypic analyses of a diverse panel revealed extensive allelic variation shaped by geographic origin and breeding history. GWAS identified major MTAs, notably on chromosomes 5A and 1B, associated with freezing tolerance and gluten strength, respectively. Genomic prediction combined with diversity-aware selection indices enabled the identification of elite parental combinations, resulting in measurable genetic gains across simulated generations, particularly for freezing tolerance and protein content. Although some quality traits, such as gluten index, showed antagonistic responses, the framework allows dynamic adjustment of selection indices to balance multiple breeding objectives. Validation of GWAS-derived KASP markers in BC_2_F_2_ populations demonstrated the effective deployment of marker-assisted selection at early generations. Overall, this framework provides a scalable and transferable strategy for integrating climate adaptation and quality traits in durum wheat breeding, with broader applicability to other complex traits under climate-driven production constraints.

## Data Availability

The datasets presented in this study can be found in online repositories. The names of the repository/repositories and accession number(s) can be found in the article/**Supplementary Material**.

## Supplementary material

Supplementary Table 1 Analysis of molecular variance of the 246 accessions based on a hierarchical structure.

Supplementary Table 2 Descriptive statistics for agronomic and quality traits measured in 2020–2021.
