# Supplementary figures and images for: Predictive breeding and marker-assisted selection for grain quality and freezing tolerance in durum wheat
Source: Front Plant Sci. 2026 Mar 10;17:1739121. doi: 10.3389/fpls.2026.1739121 (PMC13012012; doi:10.3389/fpls.2026.1739121)

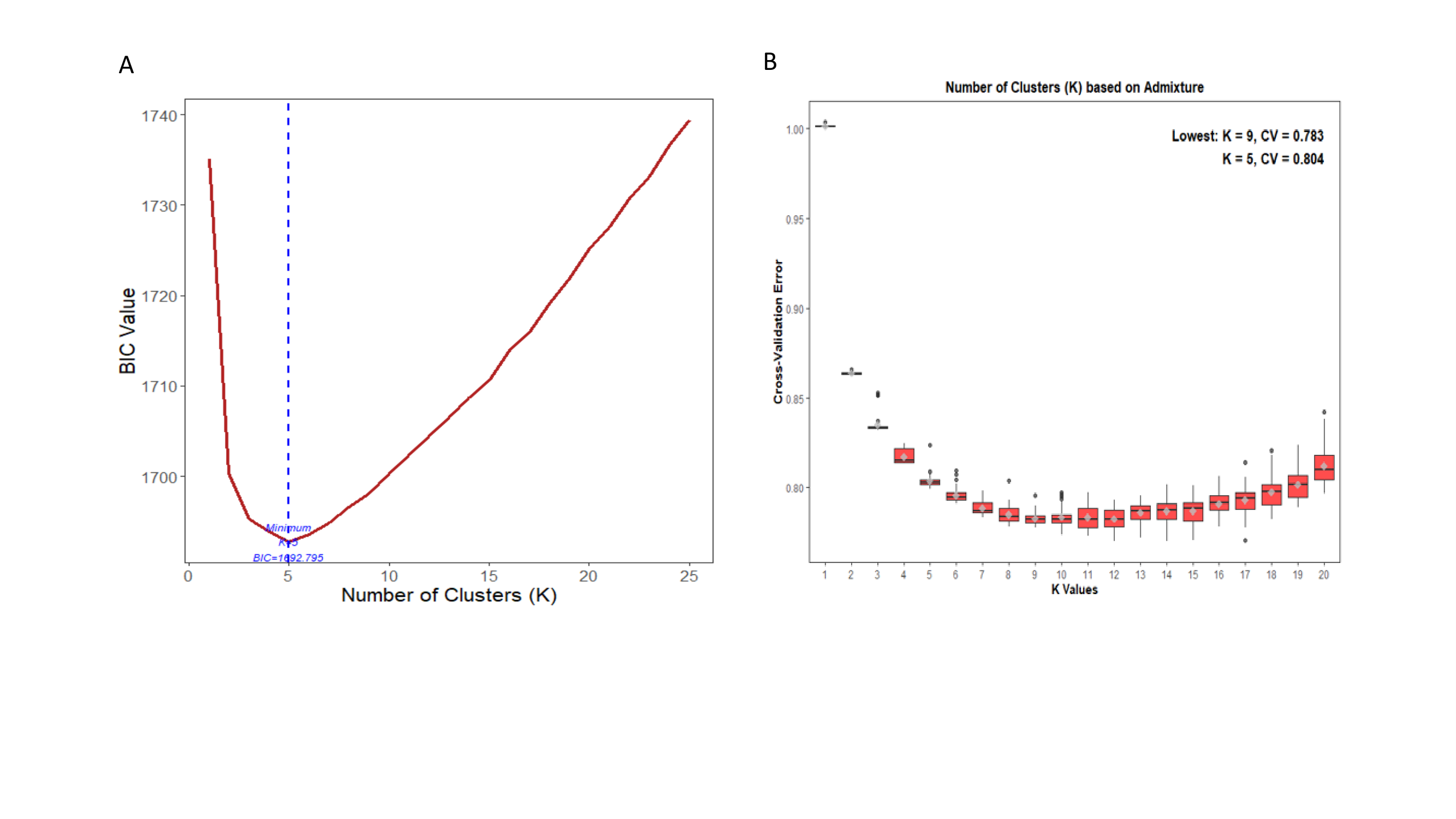

Supplement: Supplementary Table 1 — Analysis of molecular variance of the 246 accessions based on a hierarchical structure. [file Image1.tiff]

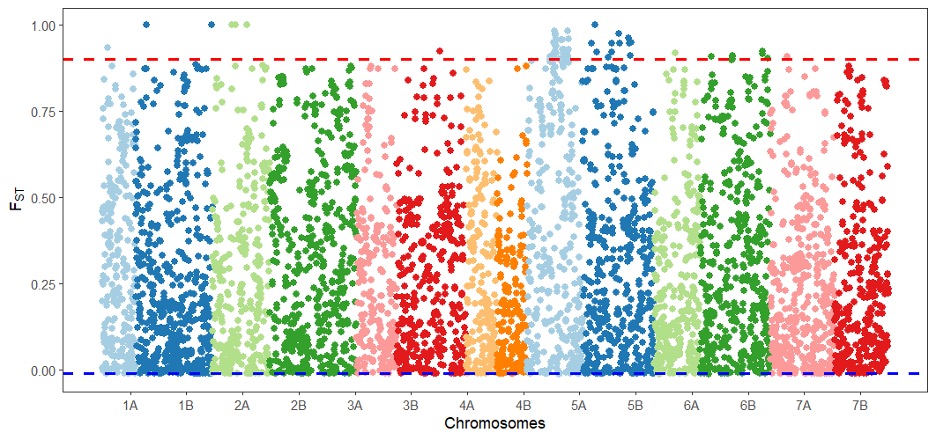

Supplement: Supplementary Table 2 — Descriptive statistics for agronomic and quality traits measured in 2020–2021. [file Image2.jpeg]

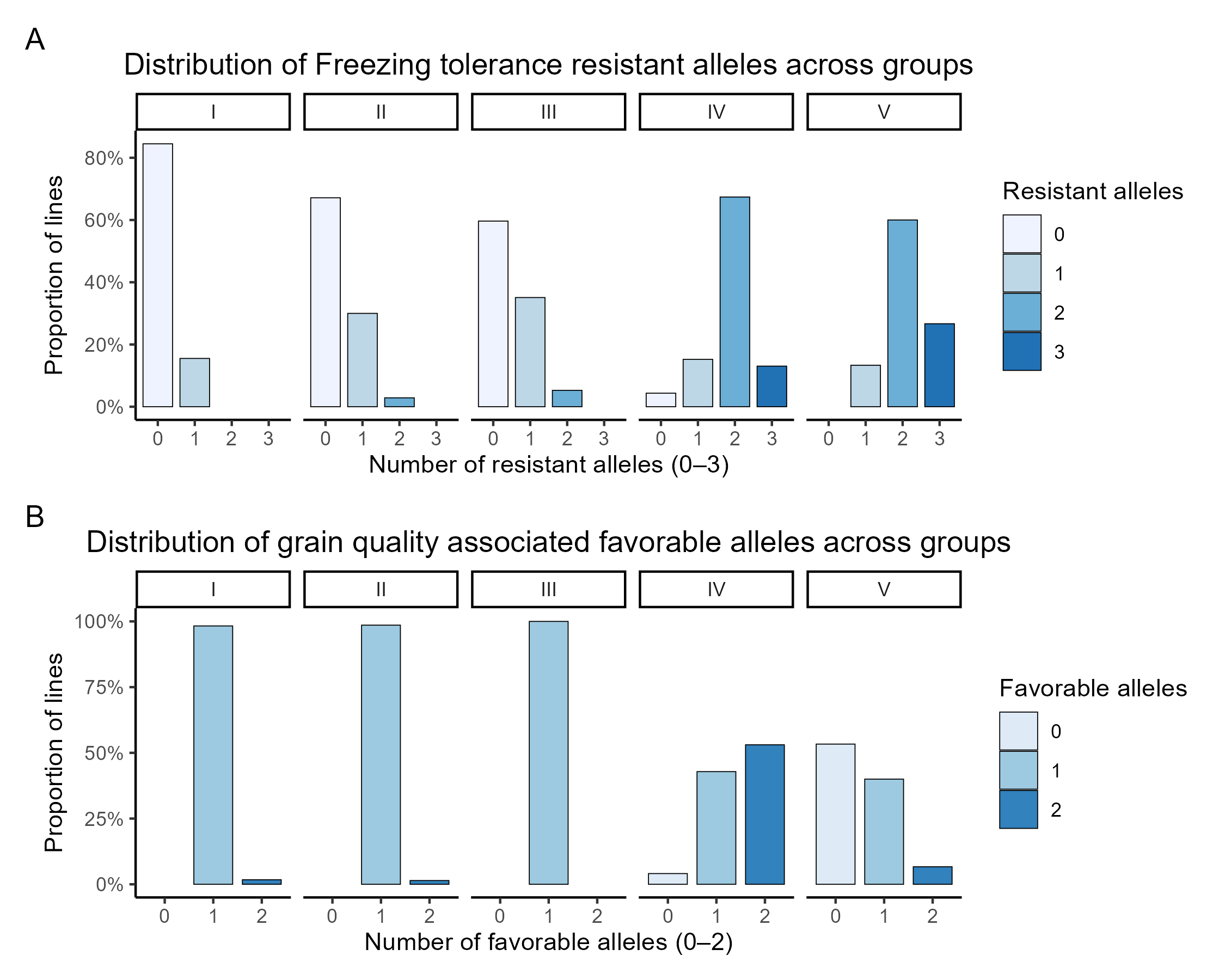

Supplement: Supplementary file 5 [file Image5.tiff]

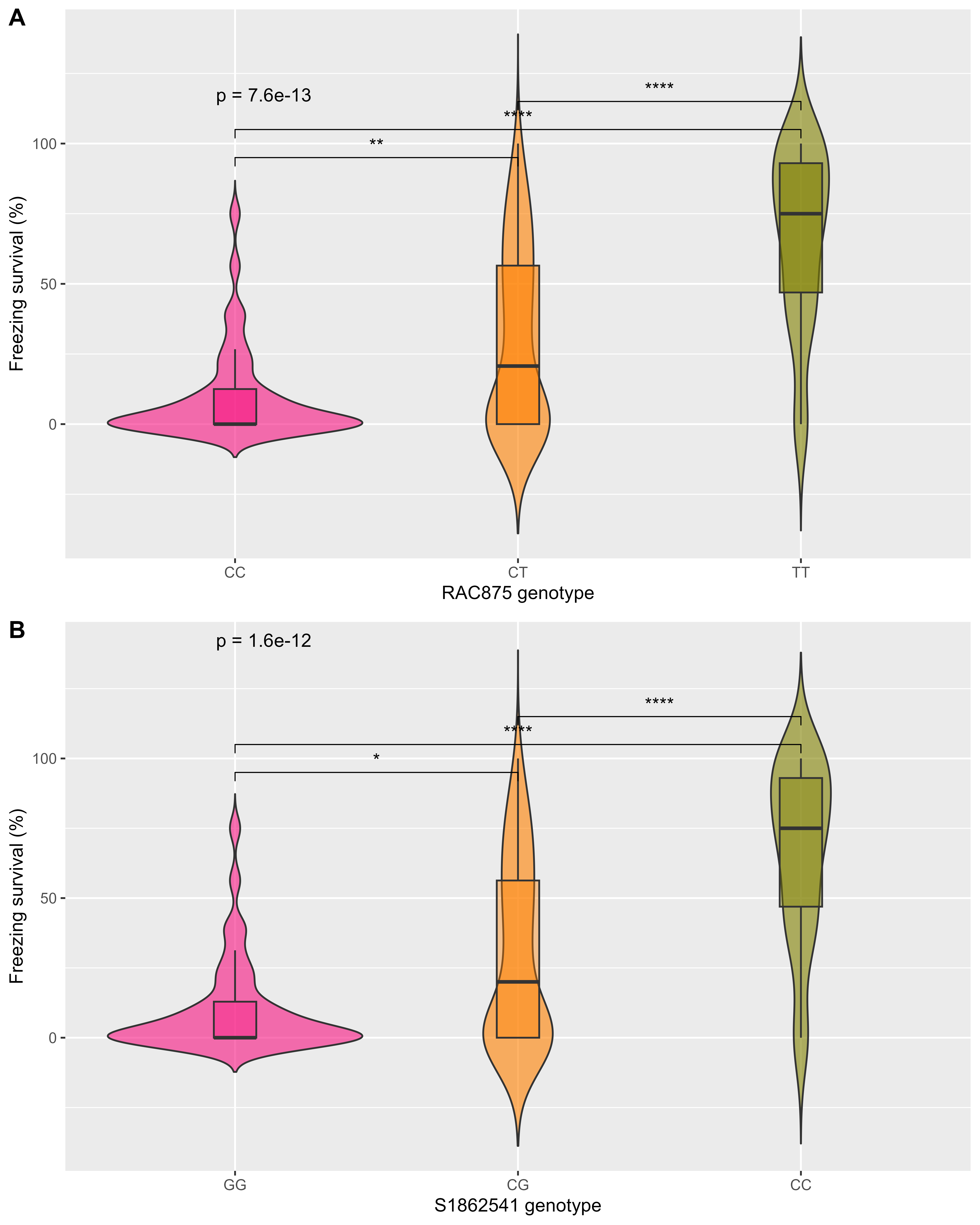

Supplement: Supplementary file 6 [file Image6.tiff]
